# Isolation and Characterization of Aerobic and Facultative Anaerobic 17β-Estradiol Degrading Bacteria in Paddy Soils and Their Potential Mechanisms

**DOI:** 10.3390/toxics13040292

**Published:** 2025-04-10

**Authors:** Wenxin Li, Shuo Yang, Hanye Ju, Chunyu Wang, Huike Ye, Xiaodong Ma, Yaqiong Wang, Mohan Bai

**Affiliations:** 1 School of Ecology, Environment and Resources, Qinghai Minzu University, Xining 810007, China; liwenxin082@163.com (W.L.); maxiaodongthu@foxmail.com (X.M.); 2 Tianjin Key Laboratory of Marine Resources and Chemistry, Tianjin University of Science and Technology, Tianjin 300457, China; yangshuo0109@163.com; 3 Agro-Environmental Protection Institute, Ministry of Agriculture and Rural Affairs, Tianjin 300191, China; juhanye@163.com (H.J.); wangchunyu6989@163.com (C.W.); yehuike@caas.cn (H.Y.); 4 Beijing Key Laboratory of Biodiversity and Organic Farming, College of Resources and Environmental Science, China Agricultural University, Beijing 100193, China; 5 Qinghai Provincial Key Laboratory of High-Value Utilization of Characteristic Economic Plants, Xining 810007, China; 6 Qinghai Provincial Biotechnology and Analytical Test Key Laboratory, Xining 810007, China

**Keywords:** 17β-estradiol, degradation bacteria, diversity, genomics, paddy soil

## Abstract

17β-estradiol (E2) contamination resulting from the widespread use of animal manure poses a new threat to the agricultural environment. Since anaerobic environments have been reported to significantly extend the persistence of E2, estrogen pollution of anaerobic farmland soils (e.g., paddy soils) is of particular concern, necessitating the development of in situ high-efficiency E2 bioremediation microorganisms. In this work, six E2-degrading strains were isolated from paddy soils, including strains from *Elizabethkingia*, *Stenotrophomonas*, *Microbacterium*, *Ochrobactrum*, *Gordonia*, and *Acinetobacter*. Among these strains, *Ochrobactrum* sp. AEPI-SP11 and *Acinetobacter* sp. AEPI-SP17 were able to degrade over 90% of 20 mg/L E2 within 5 days. Although both AEPI-SP11 and AEPI-SP17 exhibited strong tolerance to pH, temperature, and initial E2 concentrations (2, 5, 20, and 50 mg/L), only AEPI-SP17 was capable of biodegrading E2 under anaerobic conditions. Based on genomic analysis, we further obtained the whole genome sequences of AEPI-SP11 and AEPI-SP17 and identified and compared potential genes responsible for estrogen degradation in the two strains. Overall, this work significantly enhances our understanding of E2-degrading strains in paddy soils, offers valuable insights into the degradation mechanisms under varying conditions, and provides potential microbial resources for the effective control of E2 pollution in farmlands.

## 1. Introduction

17β-estradiol (E2) is the most active natural steroidal estrogen (NSE) and powerfully perturbs endocrine system, as well as having substantial ecological consequences when released into the environment [1,2]. Animal waste is the primary source of environmental E2 contamination, accounting for more than 90% of total environmental E2 contamination [3]. High concentrations of 17β-estradiol (E2) have been widely detected in manure from dairy cattle, swine, and poultry, with maximum concentration exceeding 1.2 mg/kg [4]. With the increasing utilization of manure in agricultural production, E2 pollution conferred by manure has become a substantial risk to both soil and human health. Since an anaerobic environment has been reported to extend the persistence of E2 [5,6], estrogen pollution of anaerobic farmland soils (e.g., paddy soils) is of particular concern. For instance, a survey of NSE concentrations in soils from agricultural production areas in China found that the average E2 concentration in paddy soils was approximately two times higher than that in dryland soils, with estimated risk quotient (RQ) values frequently exceeding the safety threshold [7]. Consequently, E2 pollution of paddy soil has emerged as a new and urgent issue requiring highly effective treatment.

Compared to physical or chemical treatment methods, microorganism-based bioremediation represents a significant cost advantage and is better suited for application in paddy soils under both aerobic and anaerobic conditions [8]. Previous studies have identified a substantial number of strains possessing E2-remediation capabilities. For instance, Zhao et al. [9] isolated a bacterium from a sewage treatment plant that demonstrated the ability to degrade E2, subsequently identifying it as *Serratia nematodiphila* DH-S01. Following 4 days of bacterial culturing, the degradation rate of 15 mg/L E2 by *Serratia nematodiphila* DH-S01 reached 93.2%. Similarly, Ye et al. [10] isolated *Rhodococcus* sp. P14 from mangrove sediment, which exhibited the capacity to utilize E2 as a sole carbon source and convert it into the less toxic metabolite estrone (E1). Furthermore, some bacteria were found to biodegrade E2 in anaerobic environments. Ke et al. [11] identified the degradation potential of *Agromyces* LHJ3 towards estrogen under anaerobic conditions. Notably, most of the known E2 biodegradation strains have been isolated from aquatic environments (e.g., sediment and wastewater). Comparatively, remediation strategies based on indigenous farmland degrading bacteria show great advantages due to their high environmental adaptability and low ecological risk. However, to date, few studies have paid attention to the isolation of E2 biodegradation strains from paddy soils.

In this study, we aimed to isolate and identify E2-degrading bacteria from paddy field soil that had been continuously amended with livestock and poultry manure fertilizers. A total of six strains with E2 removal capability were isolated, and two of them (AEPI-SP11 and AEPI-SP17) were found to be highly efficient. Then, we investigated the effects of various environmental conditions (pH, temperature, substrate concentration, and oxygen) on the E2 removal efficiency of AEPI-SP11 and AEPI-SP17. Based on whole-genome sequencing, the potential E2 removal mechanisms of AEPI-SP11 and AEPI-SP17 were further investigated and identified. Overall, this study is expected to improve our understanding of E2-degrading strain diversity, as well as provide germplasm resources for microbial remediation of E2 in paddy soils.

## 2. Materials and Methods

### 2.1. Sampling and Medium Chemicals

Soil samples were collected from a long-term manure-amended paddy soil in Tianjin, China. 17β-estradiol (E2), peptone, yeast extract, and sodium chloride were sourced from Macklin (Shanghai, China). HPLC-grade acetonitrile was procured from Fisher (Houston, TX, USA). All other chemical reagents and solvents employed in this study were of analytical-grade purity. The Enrichment Medium (EM) for strain isolation consisted of 1.0 g/L peptone, 10.0 g/L NaCl, and 0.5 g/L yeast extract, with the pH adjusted to 7.0. To prepare the solid agar medium, 18 g/L of agar was added to the liquid medium.

### 2.2. Enrichment, Isolation, and Identification of E2 Removal Strains

Initially, 5 g of paddy soil samples was introduced into a 150 mL Erlenmeyer flask containing 50 mL of EM liquid medium. For aerobic enrichment, the flask was incubated in a dark environment at 30 °C with a shaking speed of 180 rpm for 24 h. Subsequently, 10% of the volume of the resulting culture was aspirated and inoculated into fresh EM liquid medium, which was supplemented with 1 mg/L E2 to serve as enrichment medium. The culture conditions were maintained for 7 days (30 °C, 180 rpm), then the bacteria were transferred to fresh EM liquid medium with higher E2 concentrations (the inoculum was 10% *v*/*v*): 1, 10, 20, 50, or 100 mg/L. After enrichment, the bacterial solution was serially diluted and spread onto EM agar plates supplemented with 20 mg/L of E2. Single colonies were selected based on their growth characteristics on the EM agar plates. Multiple plate streaking was then employed for separation and purification of the bacteria.

The biodegradability of E2 by the isolated strains was investigated after a 5 day culture period. The cells were harvested by centrifugation (5000 rpm, 5 min, 4 °C), washed twice, and inoculated into EM medium with 20 mg L^−1^ E2 at a 10% ratio (*v*/*v*). The culture temperature was 30 °C, and the shaking speed was 180 rpm. The control group contained no degrading bacteria. The E2 residue in the medium was extracted with ethyl acetate. E2 was detected using an ultra-performance liquid chromatography (UPLC) system with an ACQUITY UPLC BEH C18 column (2.1 × 100 mm, 1.7 μm, Waters). During the E2 detection process, the column temperature was maintained at 30 °C, and the UV detection wavelength was set at 200 nm. The mobile phase consisted of an equal-volume mixture of acetonitrile and 0.1% phosphoric acid solution, with the flow rate adjusted to 0.2 mL/min. The efficiency of E2 degradation was determined by calculating the quantity lost based on the standard curve established for E2 quantification.

Once the ability of the strains to remove E2 was confirmed, their DNA was extracted using a Bacterial DNA Kit (Omega, Norcross, GA, USA), and the 16S rRNA gene was amplified by PCR using the universal primers 27F (5′-AGAGTTTGATCATGGCTCAG-3′) and 1492R (5′-GGTTACCTTGTTACGACTT-3′). The 16S rRNA gene sequences of strains AEPI-SP5, AEPI-SP6, AEPI-SP8, and AEPI-SP16 were submitted to GenBank and assigned the following accession numbers, respectively: PV404791, PV404792, PV404793, and PV404794.

### 2.3. Study of Removal Properties

To investigate the environmental suitability of the two highly efficient E2 removal strains isolated in this study (AEPI-SP11 and AEPI-SP17), cells of these two strains were harvested (the volume of inoculation was 10% *v*/*v*) and cultured at various pH values (5.0, 6.0, 7.0, 8.0, 9.0, and 10.0), temperatures (20, 30, and 40 °C) [5], and initial E2 concentrations (2, 5, 20, and 50 mg/L). We also performed anaerobic culture of these two strains to detect their E2 removal efficiency in an anaerobic environment. In the anaerobic experiment, 150 mL serum bottles were loaded with 50 mL of N_2_-purged EM medium supplemented with 20 mg/L of E2. All inoculation steps were conducted in an anaerobic glove box (O_2_ concentration < 1 ppm), and the inoculated serum bottles were subsequently incubated in the anaerobic chamber of the glove box. All treatments were conducted in triplicate for 5 days in the dark, and the mixtures were collected at 0, 1, 2, 3, and 5 days for E2 concentration detection.

### 2.4. Whole-Genome Sequencing and Annotation

The whole genomes of the strains AEPI-S11 and AEPI-SP17 were sequenced to reveal the genetic basis for their E2 removal adaptability. Sequencing was performed by Magigene Biotechnology Company in Shanghai, China, using an Illumina Hiseq 2000 platform (Illumina, San Diego, CA, USA). SOAPdenovo 2.04 was used for de novo assembly of the sequences, and GapCloser 1.12 was used to fill the gaps that emerged during the assembly process. Glimmer 3.02, Prodigal 2.6.3, and GeneMarkS 4.3 were used to predict coding sequences (CDS) and plasmid genes. Gene annotation was performed using the GO (version 20230830), KEGG (version 20230830), COG (version 2020.06), Non-Redundant Protein (version 20230830), Swiss-Prot (version 202312), and Pfam (version 36) databases. The obtained complete genomic sequences of AEPI-SP11 and AEPI-SP17 were submitted to NCBI GenBank under the accession number sPRJNA1213272 and PRJNA1213271, respectively.

## 3. Results

### 3.1. Isolation and Preliminary Screening of E2-Degrading Bacteria

**Figure 1:**
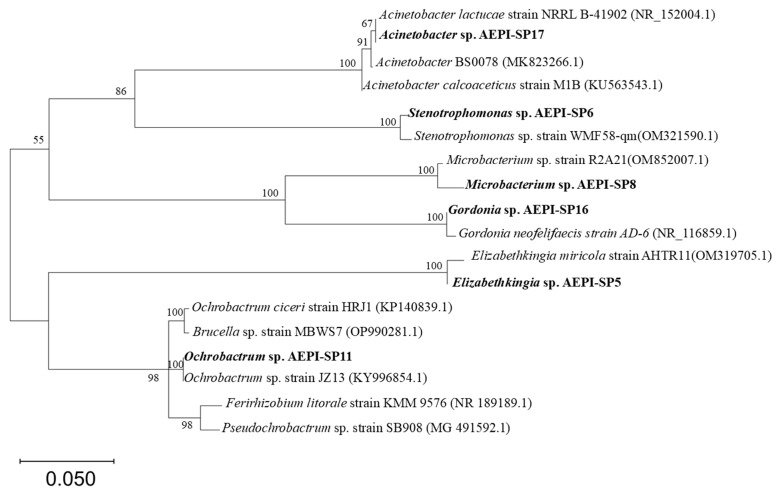
*Ochrobactrum* sp. AEPI-SP11 and *Acinetobacter* sp. AEPI-SP17 16S phylogenetic tree.

Through isolation and identification procedures, six strains capable of degrading estrogen were identified. Based on cell observation and phylogenetic analysis, these strains were classified as *Elizabethkingia* sp. AEPI-SP5, *Stenotrophomonas* sp. AEPI-SP6, *Microbacterium* sp. AEPI-SP8, *Ochrobactrum* sp. AEPI-SP11, *Gordonia* sp. AEPI-SP16, and *Acinetobacter* sp. AEPI-SP17 (Figure 1). After 5 days of culture, all strains demonstrated significant E2 degradation capabilities, with removal rates exceeding 40%, significantly higher than that of the control group (CK, 3.32% ± 0.52%). Specifically, *Elizabethkingia* sp. AEPI-SP5, *Stenotrophomonas* sp. AEPI-SP6, *Microbacterium* sp. AEPI-SP8, and *Gordonia* sp. AEPI-SP16 achieved removal rates of 55.21% ± 0.89%, 44.72% ± 1.63%, 71.44% ± 6.33%, and 72.4% ± 4.17%, respectively (Table 1). Notably, *Ochrobactrum* sp. AEPI-SP11 and *Acinetobacter* sp. AEPI-SP17 exhibited the highest degradation efficiencies, with removal rates of 93.4% and 95.4%, respectively, when exposed to an initial E2 concentration of 20 mg L^−1^ (Table 1). Additionally, the degradation process was accompanied by the generation of E1, a low-toxicity dehydrogenation metabolite. Due to their superior performance, *Ochrobactrum* sp. AEPI-SP11 and *Acinetobacter* sp. AEPI-SP17 were selected for further investigation.

### 3.2. Impact of Diverse Environmental Parameters on E2 Degradation

**Figure 2:**
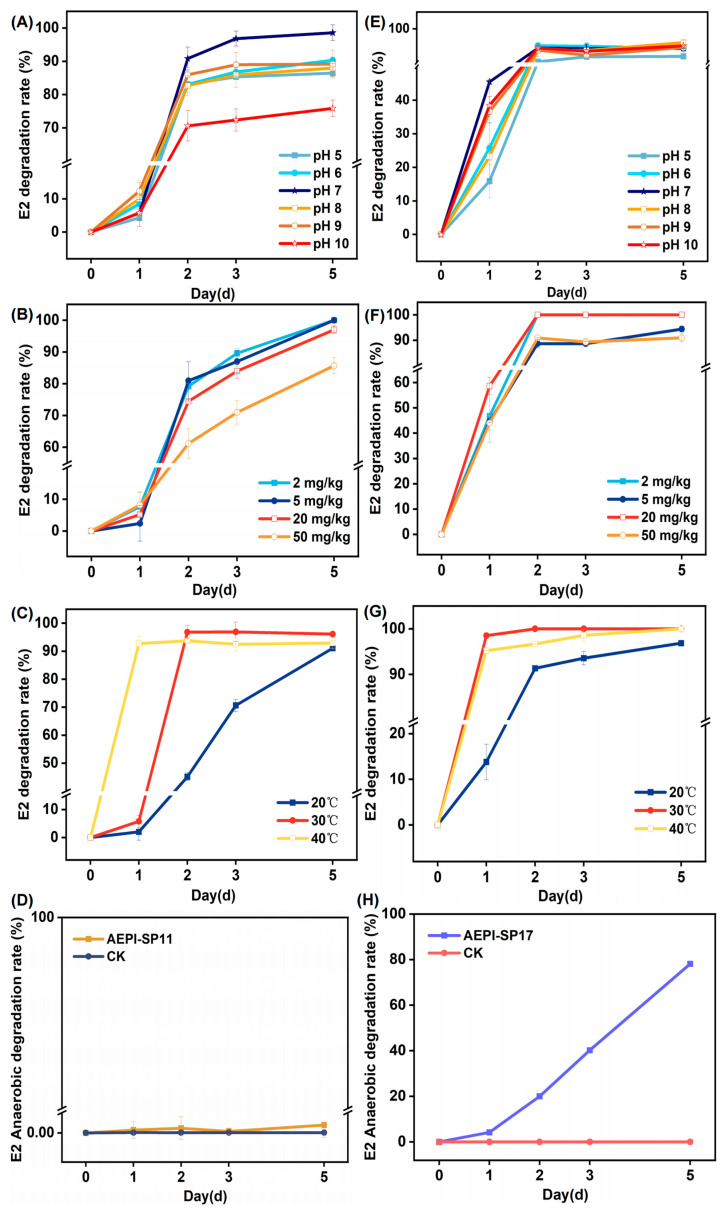
The impact of pH, initial concentration, temperature, and anaerobic conditions on the E2 removal efficiency of *Ochrobactrum* sp. AEPI-SP11 (**A**–**D**) and *Acinetobacter lactucae* sp. AEPI-SP17 (**E**–**H**). The initial concentration of E2 was 20 mg/kg in each treatment. For the anaerobic culture conditions, the temperature and pH levels were set to 30 °C and 7, respectively.

As shown in Figure 2A,E, both AEPI-SP11 and AEPI-SP17 demonstrated an optimal pH range of 7–8 for degradation. Under conditions where the pH was maintained at 7–8, with the initial concentration of the pollutant and other environmental factors held constant, the degradation rates of AEPI-SP17 and AEPI-SP11 were quantified over a specified time interval. The results indicated that strain AEPI-SP11 achieved a notably high degradation rate of 98.57% within 5 days, whereas strain AEPI-SP17 attained a degradation rate of 94.22%. The degradation efficiency of AEPI-SP11 under varying pH conditions revealed a pronounced sensitivity to pH fluctuations. Specifically, the degradation rate of AEPI-SP11 was markedly reduced in both acidic (pH 5–6) and alkaline (pH 9–10) environments compared to the optimal pH condition (pH 7) (Figure 2A). In contrast, AEPI-SP17 exhibited a more gradual variation in degradation rate across different pH levels, without the significant fluctuations observed for AEPI-SP11. This comparative analysis highlights the heightened pH sensitivity of AEPI-SP11, while the degradation performance of AEPI-SP17 remained relatively stable across a broader pH spectrum.

When the initial concentration of E2 was systematically varied, the degradation performance of AEPI-SP11 (Figure 2B) and AEPI-SP17 (Figure 2F) did not exhibit significant fluctuations. Even when the maximum added concentration of E2 reached 50 mg/kg, the degradation rate of AEPI-SP17 was still 90.92% within 5 days. As for the AEPI-SP11 strain, when the maximum added concentration of E2 was 50 mg/kg, its degradation rate reached 85.67% within 5 days. When comparing the degradation rates, although both AEPI-SP11 and AEPI-SP17 exhibited relatively stable degradation performance under different initial concentrations of E2, the degradation rate of AEPI-SP17 was consistently higher than that of AEPI-SP11. Over a temperature range of 20–40 °C, both AEPI-SP11 and AEPI-SP17 exhibited a high degradation effect on E2. At 30 °C, both strains reached their peak degradation efficiencies. The removal rates of E2 by AEPI-SP11 and AEPI-SP17 were 92.91% and 100%, respectively (Figure 2C,G).

Notably, AEPI-SP17 has the capacity for anaerobic degradation of E2. During a 5 day experimental period, we investigated the E2 degradation capacity of strain AEPI-SP17 under anaerobic conditions. The degradation rate of E2 by AEPI-SP17 reached 72.83% within 5 days. In contrast, with AEPI-SP11, the concentration of residual E2 showed almost no change, which was far lower than the E2 degradation capacity of AEPI-SP17 (Figure 2D,H).

### 3.3. Genomic Analysis of the Strains

**Figure 3:**
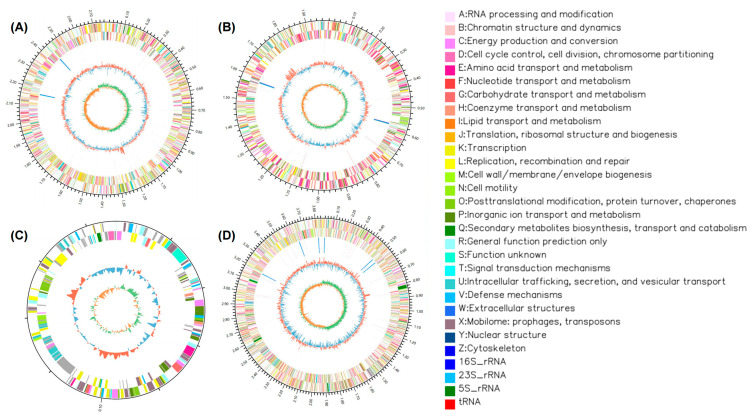
Genome sequencing plots of the strain *Ochrobactrum* sp. SP11 (chromosome 1, (**A**); chromosome 2, (**B**); plasmid, (**C**)) and the strain *Acinetobacter_lactucae* sp. SP17 (chromosome, (**D**)).

The genomic sequences of the strains were obtained through de novo assembly of the Illumina sequencing data. AEPI-SP11 possesses a genome size of 4.92 Mbp, comprising two chromosomes and one plasmid. Bioinformatic analysis predicted that AEPI-SP11 contains 19 putative estrogen-degrading enzymes encoded within its genome (Figure 3). Although the genome of AEPI-SP17 is smaller (3.85 Mbp) that that of AEPI-SP11, it encodes a higher number (27) of putative estrogen-degrading enzymes.

The functions of the obtained genes were predicted based on their annotations in the GO, KEGG, COG, Non-Redundant Protein, Swiss-Prot, and Pfam databases. The detected genes encoding particular E2-degrading enzymes were further validated by comparing them with previously reported genes in the literature [12,13,14]. Through comparison with the annotated genes, we determined that both AEPI-SP11 and AEPI-SP17 possess multiple potential polycyclic aromatic hydrocarbon–related degradation enzymes. These enzymes included quercetin 2,3-dioxygenase and catechol 2,3-dioxygenase, which are involved in catabolism of aromatic compounds, and short-chain dehydrogenases (Table 2). AEPI-SP11 exhibits a widespread distribution of steroid dehydrogenase genes across its genome, including genes encoding 3-β-hydroxysteroid dehydrogenase, 7-α-hydroxysteroid dehydrogenase, and 11-β-hydroxysteroid dehydrogenase. These genes may contribute to its notable initial capacity to degrade estradiol (Table 2). In contrast, AEPI-SP17 possesses 3α (or 20β)-hydroxysteroid dehydrogenase, along with numerous polycyclic aromatic hydrocarbon–related degradation enzymes. These enzymatic features may underlie its E2 degradation capacity.

## 4. Discussion

In this study, the isolated strains were taxonomically classified into six distinct genera: *Acinetobacter*, *Ochrobactrum, Stenotrophomonas*, *Elizabethkingia*, *Microbacterium*, and *Gordonia*. Previous studies have isolated several E2-degrading strains of *Acinetobacter* (e.g., *Acinetobacter* sp. LHJ1 [11] and *Acinetobacter* sp. DSSKY-A-001 [15]) from artificial sandy aquifers and soil. Thus, the discovery of an E2-degrading *Acinetobacter* strain from paddy soil was unsurprising. However, our study represents the first experimental evidence of anaerobic E2-degrading capacity within the genus *Acinetobacter*. Combined with its aerobic degrading nature, *Acinetobacter* sp. AEPI-S17 was identified as a facultative anaerobic E2-biodegrading strain. Indeed, previous studies have reported facultative anaerobic characteristics in some *Acinetobacter* biodegradation strains. For example, Li et al. (2022) discovered that the dibutyl phthalate (DBP)-degrading strain *Acinetobacter baumannii* DP-2 sequentially degrades DBP into dimethyl phthalate (DMP) and phthalic acid (PA) under aerobic conditions. Under anaerobic environments, *Acinetobacter baumannii* DP-2 can further convert PA into benzoic acid [16]. Since paddy soils remain anaerobic during plant growth and become aerobic between crop harvest and subsequent rice seedling transplantation, the facultative anaerobic nature of AEPI-S27 makes it well-suited for both aerobic and anaerobic bioremediation, providing a valuable microbial resource for future applications. The genus *Ochrobactrum* has been extensively documented for its bioremediation potential towards various organic pollutants, including cypermethrin [17], glyphosate [18], and petroleum hydrocarbons [19]. Furthermore, Zhang et al. [20] isolated and characterized *Ochrobactrum* sp. strain FJ1 from activated sludge, which demonstrated 98% degradation of E2 at an initial concentration of 15 mg L^−1^, within 10 days. Similarly, Ren et al. isolated *Gordonia* from mangrove sediments, which demonstrated the ability to degrade a range of E2 concentrations [21]. The genus *Stenotrophomonas* has also been isolated from wastewater and shown to exhibit remarkable E2 degradation efficiency. For example, *Stenotrophomonas maltophilia* SJTL3 was reported to degrade over 90% of 10 mg/L E2 within 1 week [22]. Our results showed that E2-degrading strains from *Ochrobactrum*, *Gordonia*, and *Stenotrophomonas* were also distributed in paddy soil, highlighting the importance of these genera in environmental E2 removal. In contrast to the previously mentioned genera, strains of *Microbacterium* and *Elizabethkingia* were identified with E2-degrading capacity for the first time, which expands our understanding of the diversity of E2-degrading bacteria in paddy soils.

Among the different strains, *Ochrobactrum* sp. AEPI-SP11 and *Acinetobacter* sp. AEPI-SP17 exhibited highly efficient E2 degradation. Even at an initial E2 concentration of 50 mg/L, AEPI-SP11 and AEPI-SP17 maintained degradation rates of approximately 85.67% and 90.92% within 5 days, which are comparable to those of previously reported highly efficient strains [11,15,20,22]. Notably, the degradation efficiency of AEPI-SP11 was significantly influenced by pH level. Compared to acidic conditions, the degradation efficiency of the strain was significantly lower in alkaline environments, which could be attributed to the fact that alkaline conditions might alter the physiological state of bacterial cells and the active sites of enzymes, thereby reducing the binding efficiency between degrading enzymes and substrates and ultimately decreasing the degradation rate [23]. Consistent with AEPI-SP11, most estrogen-degrading bacteria have been reported to achieve optimal degradation at pH 7 [23,24]. However, the AEPI-SP17 strain demonstrated remarkable adaptability to pH variations, achieving nearly complete E2 degradation within a pH range of 5.0 to 10.0 (Figure 2E). Beyond pH, temperature also played a crucial role in E2 degradation for both strains. Higher temperature was found to benefit E2 degradation for both two strains, possibly by increasing the catalytic activity of the degradation enzymes [25].

Genomic analysis identified a wide array of potential enzyme genes in the two strains, including short-chain dehydrogenases (SDRs), dioxygenases, monooxygenases, and dehydrogenases, which were previously recognized as key gene families involved in E2 removal [26,27,28]. Notably, various hydroxysteroid dehydrogenases were detected in both AEPI-SP11 and AEPI-SP17, which are considered crucial for the initial degradation of E2 and are aligned with the metabolic pathways observed in most aerobic estrogen-degrading strains [29,30,31,32]. Interestingly, although the key enzymes involved in the anaerobic degradation of estrogens remain insufficiently characterized, one study has suggested that short-chain dehydrogenases (SDRs) play an important role under anaerobic conditions [33]. In our study, a large number of SDRs were also detected in AEPI-SP17, while the key genes involved in anaerobic E2 degradation still need further research. Additionally, AEPI-SP17 possesses a greater number of genes related to polycyclic aromatic hydrocarbon (PAH) degradation, including monooxygenases and dehydrogenases. Previous studies have shown that PAHs can be metabolized into estrogenic compounds, and the chemical activation of estrogens, along with the aryl hydrocarbon receptor (AhR) signaling pathways, plays a significant role in their interaction with toxicological and metabolic processes [34]. Thus, considering the structural and metabolic process similarities between PAHs and estradiol, these PAH degradation–related genes may play important roles in E2 anaerobic degradation. However, the specific degradation pathways and mechanisms within the strains still remain largely unknown, necessitating further research using isotope-labeling techniques to detect metabolites and heterologous expression validation to study key degradation genes.

## 5. Conclusions

This study focused on the isolation, detailed characterization, and determination of the underlying degradation mechanisms of bacteria capable of degrading 17β-estradiol (E2) in paddy soils. Through a series of enrichment, isolation, and identification procedures, six estrogen-degrading strains were successfully isolated. Among them, *Ochrobactrum* sp. AEPI-SP11 and *Acinetobacter* sp. AEPI-SP17 exhibited particularly high degradation efficiencies, achieving over 90% degradation of E2 within 5 days. Notably, only AEPI-SP17 was found to possess the ability to degrade E2 under anaerobic conditions. Further genome sequencing and annotation of AEPI-SP11 and AEPI-SP17 led to the prediction of multiple potential degradation enzymes. AEPI-SP11 was found to contain steroid dehydrogenases such as 3-β-, 7-α-, and 11-β-hydroxysteroid dehydrogenase, whereas AEPI-SP17 harbored 3α (or 20β)-hydroxysteroid dehydrogenase, along with a greater number of polycyclic aromatic hydrocarbon-related degradation enzymes.

## Figures and Tables

**Table 1:** Aerobic removal rates of E2 by the E2-degrading bacteria isolated from paddy soil.

Bacterium	Degradation Rate ± SD
*Elizabethkingia* sp. AEPI-SP5	55.21% ± 0.89%
*Stenotrophomonas* sp. AEPI-SP6	44.72% ± 1.63%
*Microbacterium* sp. AEPI-SP8	71.44% ± 6.33%
*Ochrobactrum* sp. AEPI-SP11	96.11% ± 0.15%
*Gordonia* sp. AEPI-SP16	72.4% ± 4.17%
*Acinetobacter* sp. AEPI-SP17	96.85% ± 0.20%
CK	3.32% ± 0.52%

CK is the control group without degrading bacteria added.

**Table 2:** Potential E2-degradation genes in *Ochrobactrum* sp. AEPI-SP11 and *Acinetobacter_lactucae* sp. AEPI-SP17.

Category	Locus_Tag of AEPI-SP11	Locus_Tag of AEPI-SP17	Description
SDRs	gene0523, gene2386, gene3106	gene0105, gene1660, gene2526, gene0276, gene0746, gene0856, gene0950, gene1228, gene1362	short-chain dehydrogenase
HSDs	gene2019		3-beta hydroxysteroid dehydrogenase
	gene2887		7-alpha-hydroxysteroid dehydrogenase
	gene4471		11-beta-hydroxysteroid dehydrogenase
		gene3055	3alpha (or 20beta)-hydroxysteroid dehydrogenase
Di-/monooxygenases	gene0510	gene0214	quercetin 2,3-dioxygenase
	gene0389		ring-cleaving dioxygenase MhqA
	gene3388	gene0970	NAD(P)H-dependent flavin oxidoreductase
	gene3713		aromatic compound catabolic process
	gene3714	gene1645	3,4-dihydroxybenzoate catabolic
	gene0182	gene0066	catechol 2,3-dioxygenase
		gene0919	estradiol dioxygenase family
		gene1646	protocatechuate 3,4-dioxygenase activity
		gene0064	4-hydroxyphenylpyruvate dioxygenase
		gene1669, gene2535	aromatic ring-hydroxylating dioxygenase subunit alpha, beta
Other dehydrogenases	gene0007		phosphoglycerate dehydrogenase
	gene0129	gene1670	(3R)-3-hydroxyacyl-[acyl-carrier protein]
	gene0257	gene2157	enoyl-(acyl carrier protein) reductase
	gene0535	gene1808	3-oxoacyl-[acyl-carrier protein] reductase
	gene2241	gene0509	NAD(P)-dependent dehydrogenase, short-chain alcohol dehydrogenase family
	gene2477	gene1361	NADP-dependent 3-hydroxy acid dehydrogenase
	gene2386	gene3069	retinol dehydrogenase
		gene2517	2-hydroxycyclohexanecarboxyl-CoA dehydrogenase
		gene1789	2,3-dihydro-2,3-dihydroxybenzoate dehydrogenase

## Data Availability

The datasets used and analyzed during the current study are available from the corresponding author upon reasonable request.
